# Prevalence Estimate for Adjustment Disorders in the South African Navy

**DOI:** 10.2174/0117450179301661240528064329

**Published:** 2024-06-05

**Authors:** Charles H. Van Wijk

**Affiliations:** 1 Division of Health Systems and Public Health, Department of Global Health, Faculty of Medicine and Health Sciences, Stellenbosch University, South Africa

**Keywords:** Adjustment disorders, Mental health, Military, Prevalence, Risk factors, Surveys and questionnaires

## Abstract

**Introduction and Aim:**

Adjustment Disorder is among the most commonly diagnosed mental disorders in the armed forces, with a mean prevalence estimated at 7.4% across military populations. The prevalence in South African military populations is not known. This study aimed to estimate the prevalence of Adjustment Disorders in the South African Navy and to explore potential risk factors.

**Methods:**

In this cross-sectional study, a representative sample of 714 sailors completed the International Adjustment Disorder Questionnaire, and also provided information from their biennial occupational health assessment mental health screening, which included other clinical screeners of mental health and adjustment history.

**Results:**

The estimated prevalence of Adjustment Disorders in the South African Navy was 6.9%, and was proportionally distributed across gender and age categories. Depression and PTSD were the main comorbid conditions. Risk factors included a) history of adjustment difficulties during military deployment or family adjustment difficulties, b) domestic discord (difficulties in relationship with spouse/partner or immediate family), and c) the experience of stress overload (*i.e*., that the demands of life are overwhelming available resources).

**Conclusion:**

The estimated prevalence was similar to the expectation of military personnel generally, although the self-report data needs to be interpreted cautiously. The contributing risk factors offer possible direction for targeted intervention, *e.g*., skills training and relationship counselling to enhance coping with military service and family challenges, and cognitive behaviour therapy generally to address sailors’ experience of stress overload.

## 1 INTRODUCTION

### 1.1 Background

An Adjustment Disorder (AjD) is an unhealthy emotional or behavioural reaction to a stressful event or change in a person’s life and a common diagnosis within mental health treatment settings. Despite its apparent wide occurrence, surprisingly little is known about the burden of AjD in general populations [1]. An international prevalence of 2% is often cited, as is the statistic of 5-20% of all outpatient mental health visits in the US [2]. A higher burden has been described in vulnerable groups, *e.g*., 27% among the recently retrenched, up to 20% in oncology outpatient units, and 18% among bereaved individuals [3-5]. South African population prevalence is unknown.

There is a significant burden of common mental disorders (CMD) in the South African (SA) workforce [6], and the SA Navy (SAN) appears to experience a similar burden. The Institute for Maritime Medicine (IMM) provides comprehensive healthcare services to the SAN, including occupational health monitoring and mental healthcare support. Anecdotal reports suggest that high rates of AjD are present at their psychology clinics and that this diagnosis may constitute a substantial burden in the naval population. For example, during a 3-month sample period in 2022, ±45% of all new cases of naval personnel presenting at their psychology clinics were for ‘stress-response syndromes’.^1^

### 1.2 Adjustment Disorder as Clinical Syndrome

Adjustment Disorders are typically diagnosed according to one of two classification systems, namely the DSM-5 (code 309.X) and the ICD-10 (code F43.2X), or the newer ICD-11 (code 6B43). At its core, AjD can be diagnosed when 1) there is an identifiable external stressor; and 2) where an individual’s responses appear disproportionate to the stressor, and/or 3) the symptoms significantly impair functioning.

Known risk factors for AjD are low education level, being between 15 and 25 years old, being unemployed, reporting low social support, and a history of mental health disorders, and with mixed results on gender as a risk factor [7, 8]. While the literature identifies talking therapies and self-help tools as the most common treatment used for AjD in the general population [9, 10], there is currently no ‘gold-standard’ treatment that is generally recognised.

### 1.3 Screening for Adjustment Disorders

To enable cross-national application of AjD screening, the WHO developed the International Adjustment Disorder Questionnaire (IADQ) [11]. The IADQ consists of five sections, reflecting the updated ICD-11 diagnostic criteria for AjD. A probable diagnosis of AjD requires the presence of:

1) A psychosocial stressor; and

2) At least one ‘preoccupation’ symptom; and

3) At least one ‘failure-to-adapt’ symptom; and

4) Time of onset of symptom(s) within one month of the stressor; and

5) Evidence of functional impairment.

### 1.4 Adjustment Disorder in the Armed Forces

#### 1.4.1 Prevalence

AjD is the most commonly diagnosed mental disorder in the armed forces [12-14], and among USA active-duty service members accounted for 30.8% of incident mental health diagnoses in the period 2016-2020 – more than Post-traumatic Stress Disorder (PTSD), anxiety, or depression [15]. Among UK armed forces personnel, 33% of mental health consultations were associated with AjD in 2021. Further, AjD was as frequently diagnosed as depression and was seen more frequently than PTSD and substance misuse [16].

Across national samples, AjD is consistently either the most reported or second most reported mental disorder for military personnel, when compared to other commonly assessed mental disorders such as depression, anxiety, and PTSD. The prevalence estimates in general healthy military samples range from 0.7 to 16.8%, with mean prevalence estimated at 7.4% [16]. Further, among military samples with diagnosed mental health disorders, the estimated prevalence of AjD is 34.9% [16]. AjD in military samples frequently occurs comorbid with other CMDs, mainly with depression (49%) and PTSD (37%), while the association with substance abuse is not clear at present [16, 17].

#### 1.4.2 Risk Factors

Identified risk factors for AjD in military samples are 1) presence of adverse childhood experiences (ACE) [18-20]; 2) personality traits, specifically increased neuroticism and decreased extraversion [18, 19]; 3) being female (adjusted OR 1.24) [20, 21]; and previous deployment [14, 22].

#### 1.4.3 Course

About 10% of cases in military samples persist as chronic AjD, while about 25% of cases transition to another mental health disorder [22, 23]. AjD has been associated with an increased risk for separation from the military [22], but is also more likely to be associated with return to duty than other mental health diagnoses [24].

### 1.5 Aim

This study had two aims: 1) To estimate the time-point prevalence of AjD in the SAN, as well as possible comorbidities with other CMDs, and 2) To explore associations with socio-demographic variables (*e.g*., age, gender), psychological variables (psychometric scales), and general mental health and occupational adjustment history, to determine possible relative risk factors, to enhance screening and referral to appropriate services (*e.g*. mental health or social work services).

## 2 MATERIALS AND METHODS

### 2.1 Context

The IMM conducts employer mandated occupational health monitoring (which includes mental health screening) of SAN employees biennially. This study data were drawn from records of individuals assessed during 2023. IRB approval was obtained for the analysis.

### 2.2 Participants and Procedure

All sailors participating in their mandated biennial occupational health screening were invited to complete the IADQ during their occupational health assessment. Prior to giving consent and providing any information, volunteers were briefed that completion of the IADQ would not influence any subsequent health support (the IADQ was only scored after the assessments were completed). Volunteers were further offered the services of the IMM’s psychology clinics should they experience any psychological distress during or after the study period. The process was continued for a three-month sample period in 2023, during which 714 completed questionnaires were collected (94% response rate; a further 4% submitted incomplete or uninterpretable questionnaires, while 2% did not participate). This was not a sample seeking healthcare services but a general fleet sample closely representing the SAN population in terms of age, gender, and mustering. Additional data were drawn from responses to the routine mental health screening scales completed by the participating SAN personnel during their occupational health assessment.

The mean age of the 714 participants was 36.0 years (±7.9, range 21-60), and 29.6% of the sample were women. All participants self-reported as proficient in English. The sample comprised skilled workers who completed a minimum of 12 years of formal schooling with at least one year of further vocational training.

### 2.3 Measures

The IADQ was administered in its standard English form [11]. CMD’s were screened with the following scales, which were all presented in a single booklet, as per routine procedure. This was done to explore possible co-morbidities.

The *Patient Health Questionnaire-9* for depression (PHQ-9) used a score of ≥ 10 to identify cases [25, 26]. The *Generalized Anxiety Disorder questionnaire-7* (GAD-7) used a score of ≥ 10 to identify cases [26-28]. The *Primary Care Post-Traumatic Stress Disorder screen for DSM-5* (PC-PTSD-5) used a score of 5 to identify cases [29]. The *CAGE questionnaire* was used to screen for problematic alcohol use, and a score of ≥2 was used to indicate cases of concern [26, 30]. Emotional dysregulation was assessed with the *Brief Emotional Dysregulation Scale* (BEDS), which captures two components of emotional dysregulation (sensitivity and lability) and an index of direct consequences of emotional dysregulation [31].

Stress perception is an important predictor in adjustment disorders [32]. The experience of current stress overload was measured with the single item *Visual Analogue Scale for stress overload* (VAS-SO), scored on a 10-point visual analogue scale. Higher scores indicate respondents’ increased perception that the demands of their lives are overwhelming their available resources. Psychological resilience – as a possible protective factor – was measured using the *Connor-Davidson Resilience Scale-10* (CD-RISC-10) [33], with a mean of 31.3 (±4.9) found in this study.

The comorbid association of AjD and personality disorders has previously been reported [34]. Associations with pathological personality traits were thus explored using the *PID5BF+M* [35]. The 36-item self-report questionnaire represents a shortened and modified version of the original PID-5, and provides six higher-order pathological personality trait domains. The questionnaire was developed for the simultaneous evaluation of maladaptive personality traits in the DSM-5 AMPD and ICD-11 models for personality disorders. Only the six trait scores will be used here.

#### 2.3.1 Psychological History Questionnaire

As part of the occupational health assessment, participants reported their age and gender and further completed a self-report questionnaire enquiring into 1) mental health history, consisting of items with *YES/NO* answers, that enquired about previous admission to hospital or clinic for mental health concerns, and previous psychological or psychiatric out-patient treatment, 2) occupational specific adjustment history, consisting of three items with *YES/NO* answers, that enquired about general adjustment during previous deployments, difficulty with interpersonal relations in workgroup, and disciplinary issues during previous three years, and 3) family-work interface, consisting of three items with *YES/NO* answers, that enquired about risk and resilience factors during the previous three years (*i.e*., family distress, family support).

### 2.4 Data Analysis

Data were de-identified before inclusion into the study. Age was categorised into four groups, namely 20-29, 30-39, 40-49, and 50-60 years. Cases were identified as either meeting AjD diagnostic criteria or not, and reported as frequency of occurrence in the sample. Further, population estimates, using 95% confidence intervals, were calculated and reported.

Associations between categorical variables were calculated with Chi square (χ^2^) analysis, while differences in mean scores of continuous data were calculated with t-tests for independent samples. Risk factors were explored using binomial logistic regression (with AjD as dependent variable), together with receiver operating/operator characteristics (ROC) curve analyses. All statistical analyses were conducted using the Statistical Package for Social Sciences (IBM SPSS for Windows, version 29).

## 3 RESULTS

### 3.1 Prevalence Estimate

At the time of assessment, 6.9% of the sample met the criteria for an Adjustment Disorder (95%CI: 5.1% – 9.0%). Cases of AjD were distributed proportionally among women and men (χ^2^=0.024, p=.876) and across the four age categories (χ^2^=6.310, p=.097). The distribution of endorsement of psychosocial stressors is presented in Table **1**.

Further, at the time of assessment, 4.2% of the sample also met the scale threshold for Major Depressive Disorder (MDD), 1.8% for GAD, 1.2% for PTSD, and 4.9% for Alcohol Use Disorder (AUD). With reference to comorbidities: within the sub-sample of identified cases of AjD, 34.7% of participants also met the scale thresholds for MDD, 14.3% for GAD, 20.4% for PTSD, and 6% for AUD. Six cases met scale thresholds for AjD and MDD and GAD.

### 3.2 Associations with Other Variables

#### 3.2.1 Psychological Variables

There were no significant associations with histories of inpatient or outpatient psychological treatment. Significant differences in total scores on the PHQ-9, GAD-7, PC-PTSD-5, and VAS-SO were observed between individuals with AjD and those without. A significant difference was also observed for CAGE and CD-RISC-10 scores, but with smaller effect sizes and mean differences, making it less practically useful. No significant difference in BEDS subscale scores was seen, while significant differences in total scores of five of six pathological personality traits of individuals with and without AjD were found. Detailed statistical results can be found in Tables **2** and **3**.

#### 3.2.2 Occupational Adjustment Variables

Significant associations with histories of personal adjustment difficulties during previous deployments were observed but not with other markers of occupational adjustment. Detailed statistical results can be found in Table **2**.

#### 3.2.3 Family-work-interface

Self-reported histories of previous family crises that interfered with performance at work were significantly associated with the occurrence of AjD, as was self-reported domestic discord (difficulty in relationship with spouse/partner/immediate family) and self-reported unavailability of family or other social support. Detailed statistical results can be found in Table **2**.

Variables from Tables **2** and **3** with results where p<.05 were included in a binomial logistic regression. Due to substantial co-morbidity, data from the four CMDs were excluded from the regression to minimise multi-collinearity. The initial model explained 59.3% of the variance (Nagelkerke R^2^) and can be found in Table **4**. Variables with non-significant Wald tests were then removed to calculate a refined model.

In the final model, four variables that contributed to the risk for AjD were identified. Previous adjustment difficulties during deployments, a history of previous family crises that interfered with performance at work, self-reported domestic discord (recent history of difficulty in relationship with spouse/partner/immediate family), and the perception of substantial stress overload all showed an increased odds ratio for meeting diagnostic criteria for AjD. The final model explained 52.8% of the variance (Nagelkerke R^2^) and can be found in Table **4**.

## 4 DISCUSSION

### 4.1 Prevalence Estimates and Comorbidities

The 6.9% time-point prevalence of AjD, using ICD-11 diagnostic criteria contained in the IADQ, is closely aligned with the mean estimated prevalence among armed forces personnel (namely, 7.4%) [16]. However, this figure needs to be interpreted cautiously, as estimates of mental disorder prevalence are typically higher when using self-report scales [36], and personnel participating in mental health evaluations tend to more readily amplify symptoms of distress when undergoing self-report screening than when participating in person-to-person clinical interviews [37]. No gender effect was observed, supporting previous reports of proportional distribution across gender groups [8, 38].

This 6.9% estimate is higher than the 2% often cited for general civilian society and at the lower end of the 5-20% estimates of out-patient mental health visits [2]. As with other samples across countries, AjD appears to be the most common mental disorder in national armed forces [16].

Substantial comorbidities were found, though at somewhat lower rates than previous reports for military samples, for example, 49% with MDD and 37% with PTSD [16, 17]. This needs to be interpreted with caution as well, as cases of AjD (and MDD, PTSD) were determined by self-report responses to psychometric scales, and diagnoses not clinically verified. The severity of distress or impairment of functioning was not measured, and it is thus not clear whether this was true diagnostic comorbidity (*i.e*., independently present), or whether the AjD cases rather reflected a specific presentation of another, possibly more severe, condition. A quarter of AjD cases in the military evolve into more severe psychiatric disorders [22], and it could be hypothesized that the comorbidities may represent AjDs transitioning to MDD or PTSD. The substantial comorbidities emphasise the critical need for enhanced screening to facilitate timeous intervention.

The psychosocial stressor most often endorsed was concern about a loved one’s health. Within the SA Navy context, sailors typically live/work away from their province of origin, where elderly parents/grandparents still reside. The somewhat older mean age of the sample, together with the general aging and growing age-associated chronic disease burden in SA [39], would underscore concerns regarding the healthcare of aging parents/grandparents. Challenges to long-distance communication likely exacerbate concerns about older-age related health status.

Risk factors converged around previous history of adjustment difficulties during military deployment, family adjustment difficulties, presence of domestic discord, and current experience of stress overload. The association of stress overload fits into the conceptual definition of AjD. All four risk factors offer possible direction for targeted intervention, for example [40], skills training (to enhance coping with military service and family challenges), relationship counselling (to address domestic discord), and cognitive behaviour therapy (to address sailors’ experience that the demands of their lives are overwhelming their available resources). A greater allocation of resources for appropriate psychological support services might be required.

The role of psychological resilience in reducing the risk of AjD has previously been illustrated [41]. Psychological resilience in this sample – at least as measured by the CD-RISC-10 – did not appear protective of personal well-being in the presence of psychosocial stressors. However, resilience typically refers to the ability to bounce back *after* setbacks [33], and thus, the CD-RISC-10 scores may rather show the longer-term outcomes or prognosis of AjD, which was not measured in this study. Further, uniformly high scores on the CD-RISC-10 were observed in this sample. As the construct of psychological resilience is conceptually difficult to measure, the CD-RISC-10 scores may thus possibly not be an accurate reflection of participants’ actual resilience.

The presence of ACE has been identified as a risk factor for AjD in military samples [16]. While not included in the current study, this holds potential practical implications for the SA context. The role of ACE in the development and maintenance of poor long-term mental health outcomes among adults – across national, cultural, and economic groups has been extensively described elsewhere. South Africa has many young adults with a history of ACE [42, 43], and as the SAN draws new recruits from the larger SA society, the impact of ACE on adult mental health could result in increased demands for psychological services among naval personnel. In practice, the presence of AjD could alert clinicians to a history of ACE, or vice versa, which in turn could facilitate meaningful referral for intervention.

The benefits of early identification and intervention in cases of AjD and other CMDs are well described [44, 45], and military mental health policy should prioritise programs that early identify and refer to appropriate support services.

### 4.2 Limitations and Future Directions

This study used a relatively small sample accessed over a relatively short time period. The results can therefore not be generalised to other military or civilian samples without due caution. It further used self-report tools, and cases of AjD or other CMDs were not verified through clinical assessment. The limitations of a cross-sectional design are acknowledged, and longitudinal studies – to track changes over time – would be important to explore the utility of screening and intervention, as well as long-term outcomes of AjD in military personnel. Further, future studies should confirm diagnoses clinically and also consider the longer-term outcome of identified cases of AjD to determine whether any available markers would act as longer-term resilience factors.

## CONCLUSION

The estimated prevalence of AjD in this sample for the SAN was similar to the general expectation of military personnel, although the self-report data needs to be interpreted cautiously. The analysis further highlighted the role of previous history of difficulties during military deployment, family adjustment difficulties, the presence of domestic discord, and the experience of stress overload as contributing factors to a diagnosis of AjD. The results allow for mental health service provision planning, and the contributing factors might also offer possible direction for targeted intervention. This might require a greater allocation of resources to mental health support services.

## AUTHORS' CONTRIBUTIONS

It is hereby acknowledged that all authors have accepted responsibility for the manuscript's content and consented to itssubmission. They have meticulously reviewed all results and unanimously approved the final version of the manuscript.

## Figures and Tables

**Table 1:** Endorsement of categories of psychosocial stressors.

-	**Full Sample (%)**	**AjD Group (%)**
**Financial problems**	7.1	27.0
**Work problems**	3.7	16.0
**Educational problems**	4.9	21.0
**Housing problems**	4.8	25.0
**Relationship problems**	5.0	20.0
**Own health problems**	4.8	16.0
**Loved one’s health problems**	8.4	25.0
**Caregiving problems**	2.8	16.0
**Other**	2.9	16.0

**Table 2:** Association of diagnosis of adjustment disorder and related variables.

**Psychological Variables**	**χ^2^**	***p***
History of inpatient treatment	0.521	.470
History of outpatient treatment	2.531	.112
**Occupational variables**	-	-
Previous adjustment difficulties during deployments	58.149	<.001
Difficulty with interpersonal relationships in work-group	2.362	.122
Disciplinary issues at work	2.270	.137
**Family-Work-Interface**	-	-
History of family crises that interfered with performance at work (past 3 years)	98.600	<.001
Recent history of difficulty in relationship with spouse/partner or immediate family (past 6 months)	41.329	<.001
Availability of family / social support	7.568	.023

**Table 3:** T-test for independent samples for adjustment disorder and other psychometric scores.

**Scale**	***t***	***p***	**Mean Difference**	**Cohen’s *d***
PHQ-9	-15.936	<.001	6.9	2.4
GAD-7	-15.163	<.001	5.5	2.2
PC-5	-13.795	<.001	1.9	2.0
CAGE	-4.315	<.01	0.3	0.6
VAS-SO	-7.690	<.001	2.9	1.6
CD-RISC-10	2.924	<.01	2.1	0.4
BEDS-S	-1.367	n.s.	-	-
BEDS-L	-1.619	n.s.	-	-
PEDS-C	-0.531	n.s.	-	-
PID5BF+M negative affect	-6.738	<.001	2.6	1.0
PID5BF+M detachment	-5.109	<.001	2.0	0.8
PID5BF+M antagonism	-4.431	<.001	1.5	0.7
PID5BF+M disinhibition	-6.477	<.001	2.4	1.0
PID5BF+M anankastia	-1.991	n.s.	-	-
PID5BF+M psychoticism	-5.533	<.001	2.3	0.8
PID5BF+M total score	-6.346	<.001	11.9	0.9

**Note**: BEDS=Brief Emotional Dysregulation Scale; GAD=Generalised Anxiety Disorder; CD-RISC=Connor-Davidson Resilience Scale; PC-PTSD-5=Primary Care Post-Traumatic Stress Disorder screen for DSM-5; PHQ=Patient Health Questionnaire; VAS-SO=Visual Analogue Scale for Stress Overload.

**Table 4:** Binomial regression for adjustment disorder and indicated personal variables.

**Indicator**	**Wald**	**OR**	**95% CI**	**AUC**
**Initial model**				
Previous adjustment difficulties during deployments/courses	4.288*	3.75	1.07 – 13.13	.640
History of family crises that interfered with performance at work (in past 3 years)	10.152**	8.53	2.28 – 31.86	.691
Recent history of difficulty in relationship with spouse / partner / immediate family	15.189**	11.46	3.36 – 39.08	.660
Unavailability of social support	.316	1.77	.24 – 12.95	.531
Stress overload	24.406**	2.04	1.54 – 2.71	.854
Psychological resilience	.447	1.05	.92 – 1.19	.616
PID5BF+M negative affect	.102	.95	.69 – 1.30	.739
PID5BF+M detachment	1.544	.82	.59 – 1.12	.691
PID5BF+M antagonism	.902	1.19	.83 – 1.71	.662
PID5BF+M disinhibition	.042	1.03	.78 – 1.37	.749
PID5BF+M psychoticism	2.074	1.25	.92 – 1.70	.743
PID5BF+M total score	.035	1.02	.86 – 1.20	740
**Final model**				
Previous adjustment difficulties during deployments/courses	7.649*	4.42	1.54 – 12.65	-
History of family crises that interfered with performance at work (in past 3 years)	12.772**	7.90	2.54 – 24.54	-
Recent history of difficulty in relationship with spouse / partner / immediate family	16.943**	7.59	2.89 – 19.93	-
Stress overload	31.003**	1.92	1.53 – 2.42	-

**Note**: OR=odds ratio; 95% CI=95% confidence interval; AUC=area under the curve.

* = p<.01, ** = p<.001.

## Data Availability

Data supporting the findings of this study are available from the author upon reasonable request. It has not been deposited in a repository due to ethical considerations.

## Footnotes

1 Institute for Maritime Medicine, personal communication, 31 January 2023).

## LIST OF ABBREVIATIONS

ACE: Adverse Childhood Experiences
AjD: Adjustment Disorder
IADQ: International Adjustment Disorder Questionnaire
MDD: Major Depressive Disorder
PTSD: Post-traumatic Stress Disorder

## STANDARDS OF REPORTING

STROBE guidelines were followed.
